# Visualization of Pairwise and Multilocus Linkage Disequilibrium Structure Using Latent Forests

**DOI:** 10.1371/journal.pone.0027320

**Published:** 2011-12-13

**Authors:** Raphaël Mourad, Christine Sinoquet, Christian Dina, Philippe Leray

**Affiliations:** 1 LINA, UMR CNRS 6241, Ecole Polytechnique de l'Université de Nantes, BP 50609, Nantes, France; 2 LINA, UMR CNRS 6241, Université de Nantes, BP 92208, Nantes, France; 3 Institut du Thorax, UMR INSERM 915, BP 70721, Nantes, France; University of Stellenbosch, South Africa

## Abstract

Linkage disequilibrium study represents a major issue in statistical genetics as it plays a fundamental role in gene mapping and helps us to learn more about human history. The linkage disequilibrium complex structure makes its exploratory data analysis essential yet challenging. Visualization methods, such as the triangular heat map implemented in Haploview, provide simple and useful tools to help understand complex genetic patterns, but remain insufficient to fully describe them. Probabilistic graphical models have been widely recognized as a powerful formalism allowing a concise and accurate modeling of dependences between variables. In this paper, we propose a method for short-range, long-range and chromosome-wide linkage disequilibrium visualization using forests of hierarchical latent class models. Thanks to its hierarchical nature, our method is shown to provide a compact view of both pairwise and multilocus linkage disequilibrium spatial structures for the geneticist. Besides, a multilocus linkage disequilibrium measure has been designed to evaluate linkage disequilibrium in hierarchy clusters. To learn the proposed model, a new scalable algorithm is presented. It constrains the dependence scope, relying on physical positions, and is able to deal with more than one hundred thousand single nucleotide polymorphisms. The proposed algorithm is fast and does not require phase genotypic data.

## Introduction

Linkage disequilibrium (LD) refers to non-random associations of alleles at two or more loci, over the human genome [1], [2]. LD is usually present at short-range, *i.e.* for distances less than 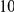 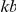 [3]. Nevertheless, long-range LD (*i.e.* LD with distances greater than 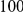 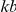) [3], and LD between different chromosomes [4], are also observed. Analyzing the extent and distribution of LD represents a major topic in statistical genetics. For instance, LD plays a fundamental role in gene mapping: the observation of a large number of genetic markers over a chromosomic region ensures a precise localization of (non-observed) causal mutations. Based on this property, genome-wide association studies (GWASs) [5], [6] aim to systematically localize causal loci over the genome using hundreds of thousands of single nucleotide polymorphisms (SNPs), an abundant and useful class of genetic markers. Beside gene mapping, LD pattern analysis offers deep insights into the understanding of human population history. Bottlenecks, natural selection and migrations are examples of evolutionary events which can be inferred using coalescent models [7].

At the interface between computer science and artificial intelligence, data mining (DM) is the process of extracting patterns from data [8]. DM helps formulate hypotheses worth testing and is complementary to more conventional statistics. Data visualization, a branch of DM, aims at providing efficient and intuitive tools to represent and summarize relevant information underlying data [9]. Data visualization has been successfully applied to bioinformatics [10].

The international HapMap project [3], and more recently the international 1000 Genomes project [11], have made considerable efforts to deeply characterize the genome sequence variation in human populations. In this context, the application of visualization methods in the analysis of LD patterns has been shown to be essential, most notably to reveal the complex so-called LD block structure [12]. The simplest but also the most popular method is the triangular heat map (THM) as implemented in Haploview [13]. The THM is the triangular matrix of pairwise dependences between genetic markers, in which the color shading indicates the LD strength in each matrix cell. The THM generally displays the Lewontin 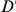 or the squared correlation coefficient 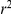. Another dependence measure, the ratio of the 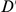 to the logarithm of odds (noted 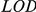), is used as a standard by Haploview. In the THM, LD blocks are visually apparent. Nevertheless, the THM has the drawback to only display pairwise dependences, thus providing a restricted view of multilocus patterns. Another popular approach consists in plotting the fine-scale map of recombination rates computed along the chromosomic sequence. For this purpose, PHASE [14], a coalescent-based method, can be used to estimate recombination rates between adjacent SNPs in the sequence. This approach helps find recombination hotspots and provides insight of the underlying block structure of LD, but leads to computational burden. More advanced techniques, such as those providing isometric blocks and bifurcation plots [15], or textile plots [16], can deal with multilocus LD. For instance, the algorithm used to draw a textile plot is closely related to principal component analysis. The textile plot strategy consists in assigning the optimal geometrical configuration to variables and data points in a low-dimensional linear space.

At the interface of graph and probability theories, probabilistic graphical models (PGMs) represent a powerful formalism to uncover complex networks of interactions. Thanks to their ability to capture (conditional) independences and dependences between variables, PGMs offer an accurate modeling of relationships between variables in an uncertain framework [17]. A PGM is a probabilistic model that relies on a graph representing conditional independences within a set of random variables. Essentially, this model provides a compact and natural representation of the joint probability distribution of the variable set. PGMs have been successfully applied to LD modeling, in particular for haplotype inference and association genetics [18]–[21]. Recently, Mourad *et al.* introduced forests of hierarchical latent class models (FHLCMs) to model genome-wide LD, together with a scalable algorithm, named CFHLC (Construction of Forests of Hierarchical Latent Class models), able to cope with 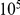 variables and 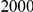 individuals [22], [23]. FHLCMs will be described in details in the next section.

In this paper, we describe another attractive property of FHLCMs (beside LD modeling) as LD visualization tools. We advocate their use for: (i) short-range, (ii) long-range and (iii) chromosome-wide LD visualization. Most notably, these models provide a compact and interpretable view of LD for the geneticist, thanks to their hierarchical graphical nature and their latent variables (LVs). Moreover, the proposed method allows to visualize both pairwise and multilocus LD on a single display, and to tackle the fuzziness of LD block boundaries. Low-level LVs represent short-range LD and are interpreted as haplotype shared ancestry, whereas high-level LVs correspond to long-range LD and are seen as population structure or natural selection effects. We also define a new multilocus LD measure for the evaluation of LD strength inside FHLCM clusters. Tested on real datasets, our method has been shown to be a valuable tool for the geneticist, regarding both information summary and understanding of LD spatial structure.

A first version of CFHLC, the learning algorithm, requires the splitting of the genome in large fixed-size windows (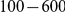 SNPs) in order to process genome-scale data. This represents a severe drawback as the possible dependences between adjacent windows are missing, which blurs the analysis of long-range LD. Thus, for an adequate use of FHLCMs in LD visualization, we have developed a new version of the CFHLC algorithm (named CFHLC+). In this second version, the genome splitting is not requested anymore to resolve the scalability issue. LD modeling is now constrained by physical position of SNPs along the chromosome. This constraint, less drastic than genome splitting, actually corresponds to a sliding window approach. Fixing the sliding window size sufficiently large (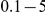 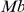) represents a reasonable strategy to capture long-range LD in the GWAS context.

This paper is organized as follows. Section Results illustrates our LD visualization approach within three experimental contexts: (i) short-range, (ii) long-range and (iii) chromosome-wide LD. The next section highlights the contribution of this paper and gives directions for future works. In the last section, Material and Methods, we explain the forest of hierarchical latent class models and its biological interpretation. Then, we present the new version of CFHLC able to learn FHLCMs from genome-wide data by constraining dependence scope using physical locations of SNPs. We also describe the new multilocus LD measure based on FHLCMs. Finally, we briefly explain the graph drawing- and display- methods used to visualize the FHLCMs.

## Results

### Short-Range Linkage Disequilibrium

We illustrate the visualization of short-range LD using the well-known Daly *et al.* dataset [12] available at http://www-genome.wi.mit.edu/humgen/IBD5/index.html. This dataset provides a good example of complex LD patterns with multiple degrees of LD. It consists of 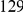 trios, each composed of two parents and one child. For each individual, 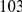 SNPs are genotyped in the 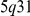 region and cover 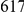 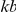.

**Figure 1:**
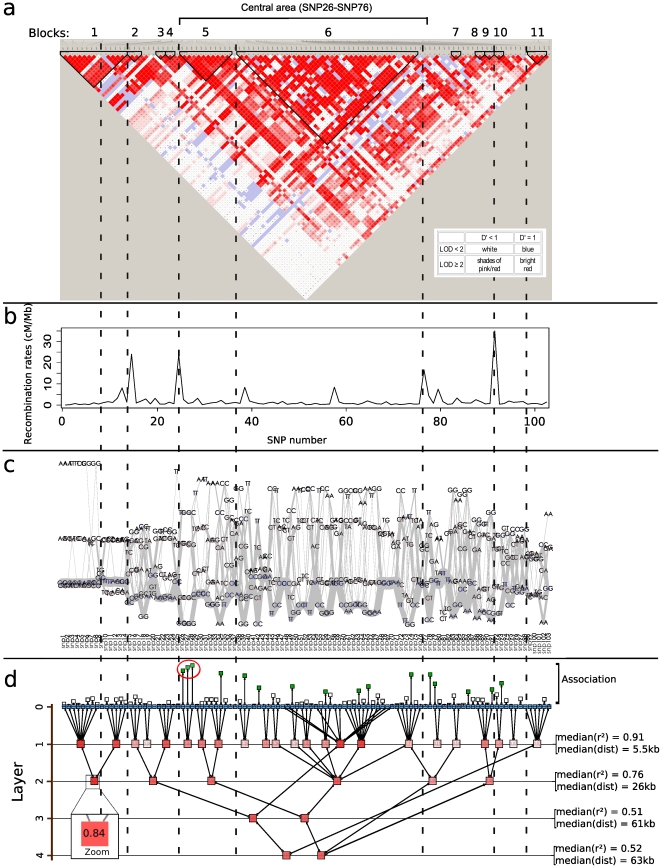
Comparison of linkage disequilibrium visualization methods applied to the Daly *et al.* dataset [**12**]. a) triangular heat map of *D′ = LOD* with LD blocks computed using Haploview v4.2, b) recombination rates inferred with PHASE v2.1, c) textile plot and d) forest of hierarchical latent class models displayed using Tulip. For each layer l, the median of *r^2^* values (resp. distances) is computed over all pairs of variables having their lowest common ancestor in layer l. The magnified node in subfigured) shows the multilocus linkage disequilibrium measure relative to the latent variable thus highlighted. Dotted lines highlight common trends between the four methods. Association signal is visualized through the length of an edge linking a SNP to an additional node.

Our FHLCM-based method is compared with: firstly two popular approaches - the triangular heat map (THM) of 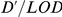 using Haploview v4.2 (www.broad.mit.edu/mpg/) and the fine-scale recombination rates inferred with PHASE v2.1 (http://stephenslab.uchicago.edu/) - and secondly the most advanced method, the textile plot (http://www.stat.math.keio.ac.jp/). Results are presented in Figure 1. In spite of the fact that these methods differently tackle LD visualization, common trends emerge: most SNPs are divided into blocks which are common between the different methods (see dotted lines). In this sequence, Haploview inferred 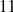 LD blocks, which are underlined in black color in the THM (see Figure 1a). Besides, we observe many dependences between blocks, most notably in the large central area SNP26–SNP76 (see bottom section of Figure 1a), between blocks 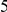 and 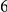. The boundary between blocks 5 and 6 is not plain. The THM also depicts strong dependences between non-contiguous SNPs, for instance, between SNPs 26 and 28 of block 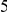 and between SNPs of blocks 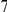, 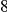, 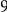 and 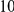. The recombination rate plot (RRP) indicates four recombination hotspots at positions SNP14–SNP15, SNP24–SNP25, SNP76–SNP77 and SNP91–SNP92, showing values beyond 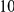 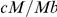 (see Figure 1b). These recombination hotspots define 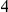 large blocks which are partly in adequacy with those obtained with the THM, as shown by the dotted lines.

In the textile plot, the greater the dispersion of the genotypes between one homozygote and the other on the vertical axis, the more likely the SNP is in LD with all other SNPs (see Figure 1c). Using the textile plot, similar results are observed compared to the THM and the RRP. Dispersions of the genotypes are high inside LD blocks and low at boundary regions. Beside the LD block view, we can distinguish the absolute LD (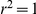 and 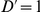), as observed between SNP1 and SNP2, from the complete LD (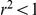 and 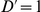), as observed between SNP2 and SNP3. For the former SNP pair, there are no connecting lines between a homozygote and the opposite side of a homozygote (*e.g.* 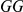 to 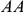 between SNP1 and SNP2), whereas there are connecting lines for the latter SNP pair. Furthermore, the textile plot offers another functionality absent in the THM and the RRP. The textile plot allows to visualize the frequencies of multilocus genotypes: the thicker the segment connecting two elementary genotypes, the higher the frequency for the corresponding two-locus genotype. For example, we observe the most frequent multilocus genotypes at the bottom section of the textile plot.

**Figure 2:**
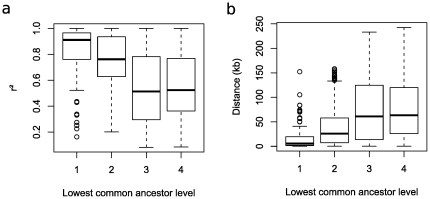
Information of the level of SNP lowest common ancestors. Relation between the level of SNP lowest common ancestors and a) the median of *r^2^* values and b) the median of distances computed over the corresponding SNP pairs. For instance, the left boxplot in a) concerns all pairs of SNPs with lowest common ancestors belonging to layer 1. Over these SNP pairs, the median of *r^2^* values is 0∶91.

The FHLCM graph provides another view of LD (see Figure 1d). This is very similar to the THM because the method also focuses on variable dependences. In the graph, leaf nodes are SNPs (blue nodes), while the other nodes are LVs (red nodes) capturing multilocus patterns. Note that the use of other nodes (white and green nodes) will be described in the second next paragraph. An edge between two nodes (latent or observed) represents the dependence between them. Thanks to the concept of lowest common ancestor for any pair of SNPs, it is possible to gain an insight of the pairwise LD strength. In graph theory, the **lowest common ancestor** (LCA) is defined between two nodes 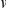 and 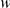 as the lowest node in the tree (or in the forest) that has both 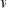 and 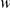 as descendants. It has to be noted that LCA is specific to graph theory and should not be confused with the most recent common ancestor used in phylogeny. In the forest, the level of the LCA related to two SNPs represents the pairwise LD strength between them. The LCA levels of SNP pairs correspond to different pairwise LD degrees and distances between the SNPs (see Figure 2). In the first layer of Figure 2, there are 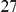 LCAs showing 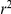 and SNP-SNP distance medians of 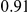 and 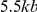, respectively. In the second layer, there are 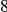 LCAs, showing 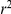 and distance medians of 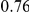 and 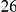 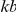, respectively. In the last two layers, 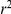 and distance medians are lower and around 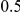 and 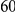 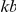. Thus, the level of an LCA in the graph brings important information on a pair of SNPs: the higher the level, the lower the pairwise LD, and the higher the distance between SNPs. Low-level LCAs represent short-range and tight pairwise LD, whereas high-level LCAs correspond to long-range and weak pairwise LD. Thus pairwise LD degrees are hierarchically displayed through LCA levels.

In addition to pairwise LD, the FHLCM graph displays multilocus LD, a complementary measure. In the graph, an LD cluster (group of non-necessarily contiguous SNPs) is easily visualized, because it is simply represented by an LV (red node) subsuming leaf nodes (blue nodes corresponding to SNPs). For each cluster, the LV color shade is proportional to the multilocus LD strength 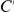. As in the THM, this representation provides a global view of LD on a single display. We observe that the distributions of pairwise LD and multilocus LD are not necessarily connected. We recall that the distribution of multilocus LD strengths corresponds to the distribution of LV shades, whereas the distribution of pairwise LD strengths can be apprehended through the levels of LCA nodes related to pairs of nodes, in the forest. Most notably, the multilocus LD strength does not depend on the LV level, contrary to pairwise LD. The multilocus LD distribution of the FHLCM is similar to the one of the textile plot, which also computes multilocus LD. For instance, the first LD block inferred with Haploview (see Figure 1a) is composed of two smaller blocks of tighter LD (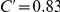 and 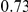, respectively). These two small blocks can easily be visualized in the textile plot and the FHLCM. The first small block shows the strongest multilocus LD in both the textile plot and the FHLCM. One asset of the FHLCM over the textile plot is that we can easily see the strong LD remaining between these two small blocks (which are both represented by an LV in layer 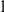), because the two small blocks are connected by an additional LV in layer 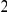. More complex dependences are observed for the large central area SNP26–SNP76, with the presence of LVs in layers 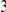 and 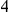. This illustrates the fact that the hierarchical nature of FHLCMs allows to easily deal with the fuzzy nature of LD cluster boundaries.

Association information can also be visualized in our plot (see Figure 1d). An association signal is represented by an edge linking a SNP node to an additional node. The length of the corresponding edge shows the strength of the signal (*i.e.* p-value). In addition, when the signal is significant (*e.g.* p-value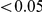), the additional node is shaded in green. In the graph, we observe a complicated pattern of multiple associations. Globally, associations are not found between contiguous SNPs, but instead they are scattered along the sequence. This is correlated with the LD structure found, which highlights numerous dependences between non-contiguous SNPs. Despite the complicated pattern, we found that the most significantly associated SNPs, *i.e.* SNP26, SNP27 and SNP28, share the same LCA in layer 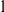 (circled in red).

Finally, we compare the number of graphical elements (NGE) between the four displays, in order to evaluate the information compactness. In the THM, the NGE equals 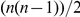, with 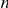 the number of SNPs. For the RRP, the NGE is 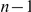, *i.e.* the number of recombination rate values (which are computed for each pair of contiguous SNPs). Regarding the textile plot, the NGE is comprised between 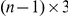 and 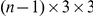, because there are 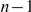 pairs of contiguous SNPs and 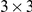 possible lines connecting genotypes (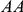, 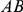 and 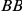) for each pair. In the FHLCM graph, NGE equals 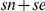, which is the sum of the number of nodes and of the number of edges. NGE varies in function of the FHLCM structure complexity. It is comprised between 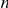 (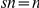, 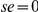) and 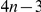 (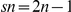, 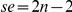). Obviously, this comparison is simplistic because it does not take into account the fact that the different methods do not provide the same amount of information. Nevertheless, it clearly demonstrates that, apart from the RRP, the FHLCM graph offers the best information compactness. Most notably, the comparison of the FHLCM graph with the most similar method, the THM, indicates that information compactness is much higher in the former (linear complexity) than in the latter (quadratic complexity).

### Long-Range Linkage Disequilibrium

#### Effects Of Natural Selection

To illustrate visualization of long-range LD due to natural selection, we have chosen the major histocompatibility complex (MHC), a large chromosomic region harboring a gene family which encodes MHC molecules. MHC molecules play an important role in the immune system and autoimmunity. Long-range LD has been reported in the MHC region [16], [24]. This can be explained by selective sweeps and population history (genetic drift), but there is also evidence indicating strong influence of recombination activity. To study long-range LD, we focused on the region 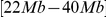 present on chromosome 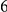 and which surrounds the MHC. Although this region contains 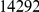 SNPs, we preferred to select only 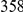 of them in the context of an off-line demonstration. It is possible to analyze all 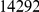 SNPs covering the MHC region but the visualization of such a large FHLCM graph requires to navigate inside the graph using software such as Tulip. We used 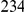 phased genotypes coming from the HapMap phase III and relative to U.S. residents of northern and western European ancestry (CEU), available at https://mathgen.stats.ox.ac.uk/impute/impute_v1.html.

**Figure 3:**
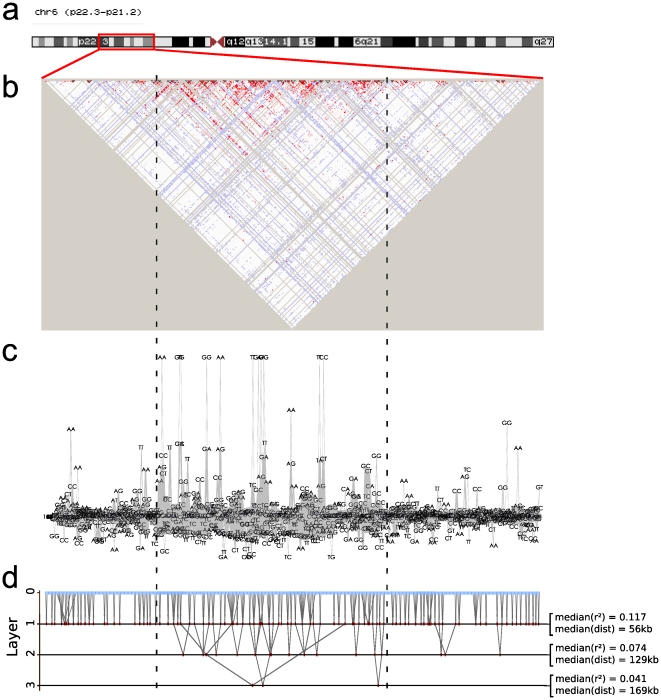
Long-range linkage disequilibrium visualization for the region [22 *Mb*–40 *Mb*], chromosome 6, surrounding the major histocompatibility complex. a) chromosome map view built with UCSC Genome Browser, b) triangular heat map of *D′ = LOD* built by Haploview v4.2, c) textile plot and d) forest of hierarchical latent class models displayed using Tulip. For each layer l, the median of *r^2^* values (resp. distances) is computed over all pairs of variables having their lowest common ancestor in layer l.

The chromosome map, the triangular heat map, the textile plot and the FHLCM graph are presented in Figure 3. The three visualization methods indicate the presence of strong LD spanning several megabases on the central area (within dotted lines), surrounded by low LD regions. In the textile plot, the large strong LD region is revealed by high dispersions of genotypes at most SNPs. In the FHLCM graph, we observe several large trees and multiple layers in the central area. Compared to the textile plot, the FHLCM graph provides additional information: its multiple layers allow to distinguish between short-range LD and long-range LD. The former is displayed through low-level LCAs while the latter is highlighted by high-level LCAs. In the FHLCM, dependences between distant SNPs are easily observed. It is not the case with the textile plot where SNPs are ordered along the chromosome. To overcome this restriction, Kumasaka *et al.* use a hierarchical clustering variable algorithm which rearranges SNP positions along the horizontal axis, allowing to show LD between distant SNPs. Nevertheless, the drawback is that physical ordering of SNPs is lost.

#### Effects Of Population Admixture

We also studied the presence of long-range LD due to population admixture. For this purpose, we chose the example of the African ancestry in Southwest USA (ASW) population from the HapMap phase III. ASW is a well-known admixed population [25]. This data sample consists of 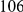 phased haplotypes. We focus on chromosome 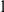 for which we selected 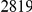 regularly spaced SNPs, in the context of an off-line demonstration. A sliding window of 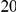 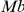 has been used.

**Figure 4:**
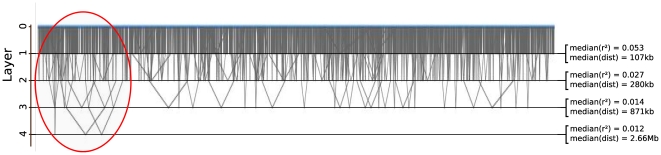
Long-range linkage disequilibrium visualization of 2819 regularly spaced SNPs of chromosome 1, for the African ancestry in Southwest USA (ASW) HapMap population. A long-range LD region is circled in red.

The FHLCM graph is plotted in Figure 4. The graph presents 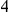 layers of LVs. LD varies from 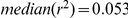 in the first layer to 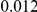 in the fourth layer. In the first layer, the median distance between SNPs is around 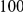 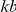, while it is 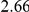 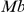 in the last layer. As observed with CMH, dependences between distant SNPs are easily observed. We are able to localize regions showing long-range LD, such as the region at the beginning of the chromosome (circled in red).

### Chromosome-Wide Linkage Disequilibrium

Chromosome-wide LD visualization can be performed by learning FHLCMs with CFHLC+ and by navigating through the corresponding graphs with Tulip. We illustrate the LD visualization of chromosome 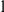 for CEU population. The dataset consists of 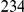 phased genotypes and 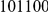 SNPs. FHLCM learning was constrained by a maximum physical distance between SNPs (or LVs) of 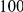 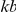. CFHLC was run on a standard PC (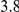 GHz, 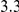 GB of RAM). Only 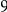 hours and 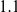 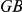 were necessary to compute the FHLCM for the entire chromosome.

**Figure 5:**
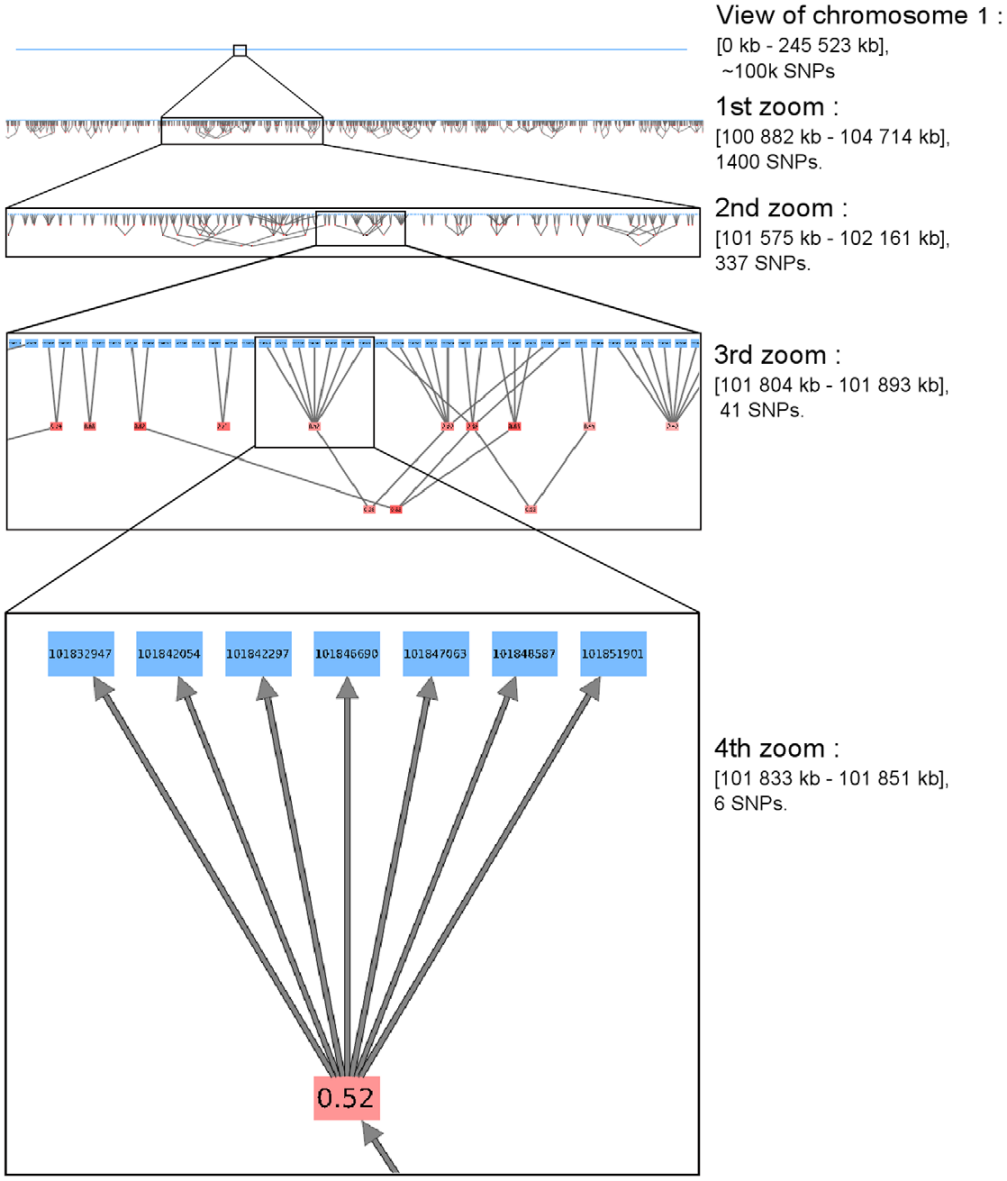
Chromosome-wide linkage disequilibrium visualization of chromosome 1. Navigation through zooming inside the FHLCM graph. Positions of SNPs are displayed inside blue nodes. The multilocus LD strengths relative to the subtrees rooted in the latent variables can be viewed inside red nodes.

In Figure 5, the FHLCM graph is depicted. Navigation in the graph through successive zooms allows to change the resolution for the visualization. When no zooming function is active, the chromosome is simply represented by a blue line. Nevertheless, if we zoom a first time on the graph, the global structure of LD becomes apparent. In the second view, long-range LD between SNPs spaced by 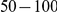 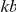 is easily visualized. In the third display, it is possible to distinguish each LD cluster in the graph (*i.e.* each FHLCM subtree), to see the number of FHLCM layers and the degree of connectivity. The degree of multilocus LD is shown by the color shade of LVs. Finally, if we zoom again, we can see the position of SNPs and the precise multilocus LD strength measure, which are written inside blue nodes (SNPs) and red nodes (LVs), respectively.

## Discussion

Our FHLCM-based method for visualization of LD spatial structure has been shown to provide a compact view of LD spatial structure in the three main contexts: short-range, long-range and chromosome-wide LD analyses. Our approach focuses on variable dependences, and thus is very similar to the THM. Beside plotting pairwise LD, the FHLCM can also show multilocus LD, which represents the most important asset. Moreover, pairwise LD degrees are hierarchically displayed by LCA levels. Compared to the textile plot, our visualization tool shows several drawbacks, but also multiple advantages. Although FHLCM graphs do not allow to distinguish between complete and absolute LD, or to show genotype frequencies, it clearly reveals long-range LD without necessitating any rearrangement of the SNP order in the sequence, such as required for the textile plot. In fact, the textile plot and the FHLCM graph are complementary approaches to study LD structure.

Future researches will focus on two main aspects. First, important information provided by FHLCMs has not been used in our visualization approach. Conditional and *a priori* probability distributions learned by CFHLC could provide insights of the frequencies of genotypes, and above all, of the frequencies of genotype clusters. Finally, the next step is providing the geneticist an integrated software tool equipped with a user-friendly interface, such as provided by Haploview or the Textile Plot software, to construct FHLCMs, display them and launch off-line genetic association analyses.

## Materials and Methods

### The Model And Its Biological Interpretation

From now on, we will restrain the study to discrete and finite variables (either observed or latent).

**Figure 6:**
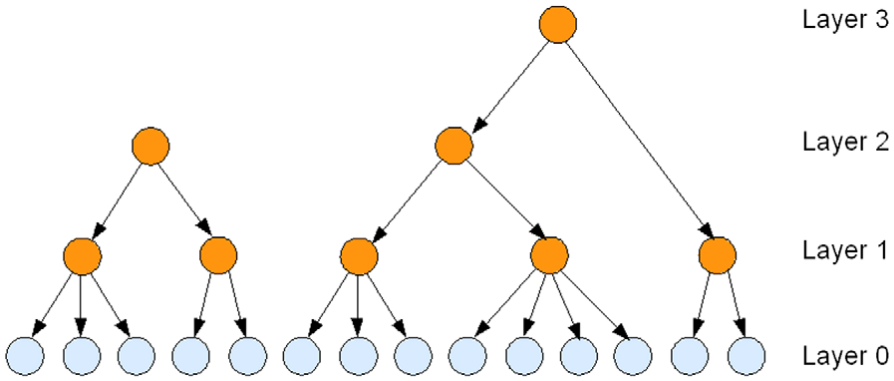
The forest of hierarchical latent class models. The light shade (blue) indicates the observed variables whereas the dark shade (red) points out the latent variables.

**Figure 7:**
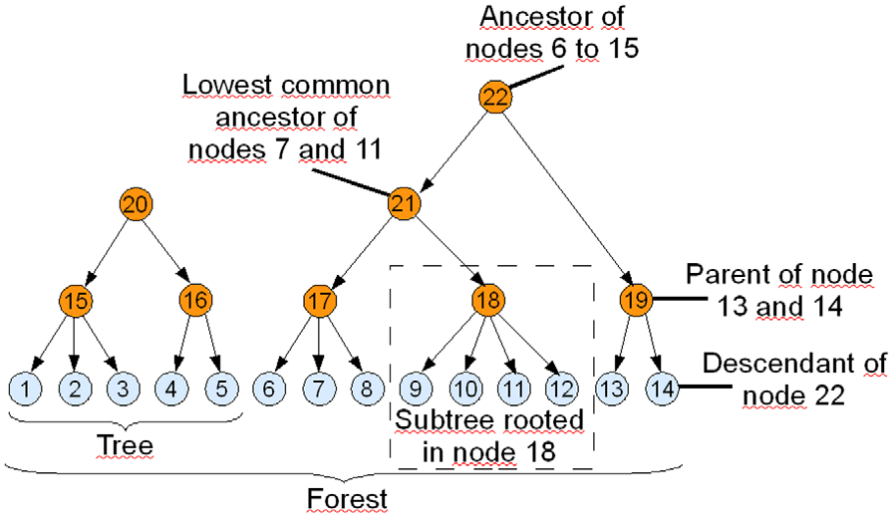
Illustration of specific key terms: subtree, tree, forest, parent, descendant, ancestor and lowest common ancestor. See Figure 6 for node nomenclature.

FHLCMs are forests whose trees are hierarchical latent class models (HLCMs). An HLCM is defined as a tree whose leaves are observed variables while internal nodes are latent variables organized in multiple layers. An FHLCM is illustrated in Figure 6. The meaning of specific key terms is clarified in Figure 7. Most notably, the benefits of using FHLCMs rely on the ability of latent layers to account for multiple degrees of SNP dependences and to naturally deal with the fuzzy nature of LD block boundaries [26]. Moreover, FHLCMs offer a generalization of the block-like structure. Inside blocks, SNPs are necessarily contiguous. For some genomic regions, the block-like structure can be irrelevant [27]. Instead of modeling blocks, FHLCMs describe clusters for which the contiguity constraint is relaxed.

In the FHLCM, LVs bring a biological meaning for the geneticist. For instance, in the case of haplotype data analysis (phased genotypes), LVs are likely to represent the haplotype cluster structure of LD. To a certain extent, an LV might be interpreted as the shared ancestry of the haplotypes defined by the observed variables, namely, the contemporary haplotypes of the tree rooted in the LV. Each state of an LV may represent a group of similar haplotypes. In the situation of limited ancestral recombination, similar haplotypes tend to share recent common ancestry [5]. Although this situation is not guaranteed along the genome, it is very likely for low-level LVs, since they are expected to cover very small genomic regions showing strong LD. Besides, when the latent variables capture dependences between distant SNPs (or distant groups of markers), they can be viewed as population structure or natural selection effects. Thus the interpretation of LVs depends on their level in the graph. Low-level LVs covering small genomic regions in strong LD represent haplotype shared ancestry. In contrast, high-level LVs which capture weak dependences between distant SNPs correspond to population structure or natural selection effects. This distinction between multiple levels allows an easy and interpretable view of LD for the geneticist.

### FHLCM Learning

In the CFHLC+ algorithm, the learning is performed through an adapted agglomerative hierarchical clustering procedure: (i) at each agglomerative step, a clique partitioning method is used to identify cliques of dependent variables (*i.e.* LD clusters); (ii) each such clique is subsumed into an LV, through a latent class model (LCM). An LCM is an HLCM which only contains one LV. For each LCM, parameter learning using the expectation-maximization (EM) algorithm and missing data imputation through probabilistic inference (for the latent variable) are performed. Iterating these two steps yields a hierarchical structure. In other words, latent variables capture the information borne by underlying observed variables (*e.g.* genetic markers). In their turn, these latent variables, now playing the role of observed variables, are summarized through additional latent variables, and so on. Details about clique partitioning, LCM-based data imputation for latent variables and LCM-based parameter learning of the hierarchical structure are described in the following.

#### Clique Partitioning Algorithm

A clique partition is a set of non-overlapping cliques of variables (cliques of variables can be seen as clusters of variables). The set of variables constituting the clique is likely to be subsumed into an LV. Overlapping clusters are proscribed since, in an HLCM, two latent nodes cannot share the same child. We applied clique partitioning to the complete graph of pairwise dependences. We used CAST [28], a clique partitioning method, and pairwise mutual information as a measure of pairwise dependence.

To deal with genome-scale data, a simple idea is implemented: pairwise dependences are only computed between variables (SNPs or LVs) which are separated by a maximum physical distance on the chromosome. Unlike SNPs, LVs do not have a physical location on the chromosome. To tackle this issue, for an LV, the average of the subsumed SNPs' locations is used. The physical constraint leads to calculate a sparse matrix of pairwise dependences, where only computed values are stored. The clique partitioning algorithm CAST has been reimplemented to handle large sparse matrices.

LD modeling is constrained by the physical positions of the SNPs along the chromosome. This constraint, less drastic than genome splitting (previously proposed in Ref. [22], [23]), actually corresponds to a sliding window approach. Fixing the sliding window size sufficiently large (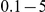 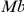) represents a reasonable strategy to capture long-range LD in the GWAS context.

#### LCM-Based Data Imputation For Latent Variables

Locally, the data imputation is achieved relying on one of the simplest Bayesian networks: we consider the LCM rooted in the latent variable and whose leaves are the variables in the clique. Then, parameter learning yields the marginal distribution of the latent variable and the conditional distributions of the child variables. Parameter learning is implemented through the expectation-maximization (EM) algorithm. Subsequently, given an individual 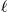 and the vector of its values 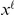 (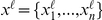) corresponding to the vector of the variables in the clique 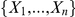, a value 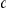 is assigned to the latent variable 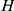 through (linear) probabilistic inference:
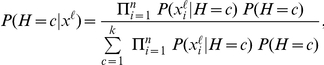
with 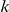 the number of classes of LV 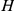. Therefore, throughout the bottom-up procedure, after the current step's completion, the newly created latent variables will, in their turn, play the role of observed variables to seed the next step.

#### LCM-Based Parameter Learning

The role of the aforementioned local LCMs is central to the FHLCM learning algorithm. Not only do they allow data imputation for the corresponding LVs but, in parallel with the structure growing, the FHLCM parameters are also learned as follows: at 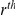 step, in addition to previously created LVs and initial OVs not already included in the hierarchy, all LVs created at 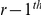 step play the role of OVs. For any such former variable shown to be a leaf node in an LCM (corresponding to a clique), the current marginal distribution is replaced with the conditional distribution learned in the LCM. Thus, during the bottom-up construction of the FHLCM, marginal distributions are successively replaced with conditional distributions.

#### Implementation

CFHLC+ can process both phased and unphased genetic data. Our algorithm has been developed in C++, relying on the ProBT library dedicated to Bayesian networks (http://bayesian-programming.org). CFHLC+ is available for Windows 32 bits at https://sites.google.com/site/raphaelmouradeng/home/programs.

### Multilocus Linkage Disequilibrium

Since FHLCMs represent multilocus LD, it is possible to compute a multilocus LD value from the joint probability distribution. Perhaps most interestingly, multilocus LD can be calculated for each FHLCM subtree (*i.e.* for each LD cluster).

Total correlation is a generalization of mutual information for multiple variables [29]. It quantifies the redundancy or dependence among a set of 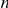 random variables 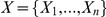. It is defined as the Kullback-Leibler divergence [30] between the joint distribution 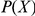 and the independent distribution 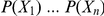:
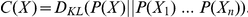
To assess multilocus LD, total correlation over SNPs is a relevant measure to evaluate the difference between the distribution assuming linkage disequilibrium (joint distribution) and the distribution assuming linkage equilibrium (independent distribution).

Total correlation is reduced to the simpler difference of entropies:
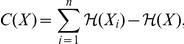
where the first term is the sum of entropies of individual variables and the last term is the entropy of the joint distribution of variables. The entropy 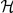 is a proper measure to assess disorder in a system 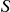 and is defined as:
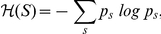
where 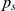 denotes the probability of each state 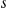 in the system 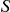, and the sum includes all possible states.

Based on total correlation, we designed a multilocus LD measure relying on the learned FHLCMs. First, we need to introduce how to compute the joint probability distribution (JPD) in general Bayesian networks (BNs). Let us consider a BN modeling a set of nodes 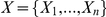, representing 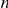 random variables. The JPD is calculated using the recursive factorization formula:
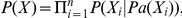
where 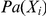 denotes the parents of node 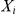, and 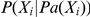 is the conditional probability distribution of 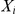 knowing 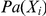.

Now we describe the computation of the multilocus LD measure in an FHLCM subtree. In the following, we only consider subtrees composed of an LV and of all its descendants, which actually corresponds to HLCMs (*e.g.* the subtree rooted in node 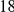 in Figure 7). Let us take for example an FHLCM subtree defined on a set of 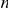 observed and latent nodes 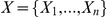. 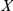 is composed of a set of 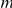 observed nodes 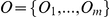 and a set of 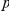 latent nodes 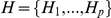. 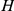 can also be divided into two subsets 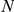 and 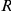: in the former, 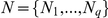, there are 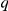 non-root nodes, whereas in the latter, 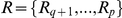, there are 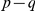 root nodes.

To compute total correlation, we replace the entropy 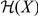, assuming linkage disequilibrium, by the entropy of the joint distribution modeled by the FHLCM subtree which is the following:
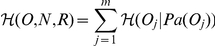


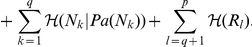
Thus, the total correlation 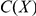 writes as:
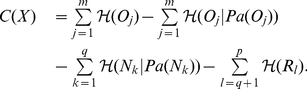
(1)We recall that mutual information, a well-known quantity to assess the dependence between two variables, can be expressed for two variables 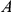 and 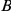 as:
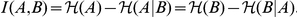
Using the mutual information formula, Equation 1 is reformulated:
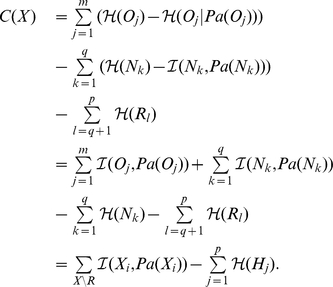
We observe that the total correlation 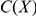 is composed of two terms: the former is often used to evaluate the fitness of a BN tree, or forest, without LVs (*i.e.* a score), whereas the latter can be seen as a penalization term specific to latent models. This penalization term is the sum of LV entropies, thus allowing to take into account the complexity increase due to the incorporation of LVs in the model. The entropy of an LV increases with both the number of its classes (*i.e.* states) and the uniformity of its distribution.

Finally, we scale 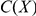:
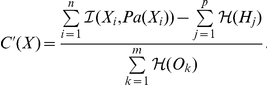
In rare situations, 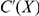 can be slightly below 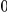 (due to the penalization term). In this case, its value is set to 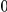. Efficient computations of 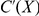 can be done starting from the bottom layer and ending at the top layer of the FHLCM. The generalization of the computation of the 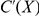 measure to a whole forest is straightforward.

The interpretation of normalized total correlation 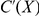 is similar to that of the 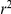 coefficient, which is used for pairwise LD. 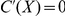 means perfect equilibrium among SNPs, whereas 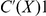 means that SNPs are in absolute LD (the SNPs provide exactly the same information). Note that because of the penalization term, the value of 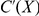 cannot be equal to 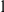. Values between 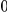 and 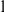 corresponds to different degrees of multilocus LD.

### Graph Drawing And Visualization

Graph drawing (*i.e.* node placement) and visualization (*i.e.* display) of FHLCMs represent an important step. For this purpose, we propose a simple method which offers a clear and interpretable view of LD spatial structure. Thanks to the hierarchical nature of FHLCMs, it is possible to implement an easy and intuitive drawing: nodes are placed along the chromosome, and layer by layer. SNPs are placed along the chromosome using their physical order on the sequence. LVs are placed using physical orders computed by averaging over the orders of the subsumed SNPs. Each layer is positioned along a line parallel to the chromosome.

Regarding graph visualization, only a few software programs have been developed to handle large graphs, such as required for genome-wide LD modeling using FHLCMs. Among others, the software Tulip (http://tulip.labri.fr/TulipDrupal/) is a user-friendly tool able to deal with about one million nodes. Together with the navigation in such large-scale graphs, including zooming in narrower and narrower regions, Tulip allows the extraction of subgraphs and the enhancement of the results thus obtained by filtering. To visualize multilocus LD for each FHLCM subtree, we propose to shade the LV node subsuming the subtree, proportionally to the LD strength (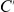). The precise value of LD is also displayed inside this LV. Association information can also be visualized. For this purpose, the association signal is represented by an edge linking a SNP node to an additional node. The length of the edge is a linear function of the 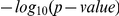 value, computed between the SNP and the phenotype. The additional node is green when the association is significant and white otherwise.
